# Effects of WeChat follow-up management on the psychological distress, care burden, and quality of life of parents of infants with bronchopulmonary dysplasia: a retrospective cohort study

**DOI:** 10.3389/fped.2023.1239527

**Published:** 2023-08-10

**Authors:** Zhong-Shan Shi, Xin-Bing Wang, Ming-Cong Wang, Yan-Yan Zeng

**Affiliations:** Department of Pediatrics, Shishi General Hospital, Quanzhou, China

**Keywords:** bronchopulmonary dysplasia, WeChat, psychological distress, care burden, quality of life

## Abstract

**Objective:**

The objective was to explore the impact of WeChat follow-up management on the psychological distress, care burden, and quality of life of parents of infants with bronchopulmonary dysplasia (BPD) receiving in-home care.

**Methods:**

This was a retrospective cohort study. A total of 101 parents of infants with BPD who were followed up from January 2016 to January 2022 were included in this study. According to different follow-up methods, these patients were classified into the WeChat group and the routine group. The Depression, Anxiety, and Stress Scale-21 (DASS-21), Zarit Caregiver Burden Interview (ZBI), and WHOQOL-BREF were used. The data on the psychological distress, care burden, and quality of life of the parents in the two groups were analyzed and compared at discharge and at the 3-month follow-up.

**Results:**

There was no significant difference in the DASS-21 and ZBI scores at discharge between the parents in the two groups. During the 3-month follow-up, the scores of the DASS-21 anxiety and stress subscale and the ZBI of parents in the WeChat group were significantly lower than those of parents in the routine group (*P* < 0.05); however, there was no significant difference in the depression subscale score between the two groups (*P* > 0.05). A comparison of the WHOQOL-BREF score between the two groups showed that the total quality of life score in the WeChat group was significantly higher than that in the routine group (*P* < 0.05). The scores of the psychological and social relationship fields in the WeChat group were significantly higher than those in the routine group (*P* < 0.05). The incidence of adverse events during follow-up was significantly lower in the WeChat group than in the routine group (*P* < 0.05).

**Conclusion:**

WeChat follow-up management is helpful to decrease the anxiety and stress, reduce the care burden, and improve the quality of life of parents of infants with BPD receiving in-home care.

## Introduction

Bronchopulmonary dysplasia (BPD) is a chronic and common disease in premature infants. The lower the weight and the younger the gestational age of a premature infant, the higher the incidence of BPD is (1). The incidence of BPD increases 2–3 times for every week of gestational age (2). In recent years, with the improvement of the medical level of neonatal intensive care units (NICUs) and maternal and infant care technology, the survival rate of premature and low-birth-weight infants has increased significantly (3, 4). Under the influence of the particular disease characteristics of BPD and the low resistance of premature infants, the disease cycle is usually long and can extend from the neonatal period to early childhood. This disease makes infants very dependent on oxygen for a long time, possibly lasting for 10–33 months. More than 30% of patients with BPD receive home oxygen therapy (HOT) after discharge (5). As a result, infants with BPD often need a long period of family rehabilitation and care. As the maintenance and management of the health of infants with BPD have high requirements, professional and meticulous care must be provided in the process of home care. As a result, parents of infants with BPD face increased stress, increased caregiver burden, and decreased quality of life, which is similar to caregivers of infants with other chronic diseases (6, 7).

WeChat is a free application launched by Tencent in China to provide instant messaging services through intelligent terminals, which has a very high penetration rate in China (8). Wang et al. showed that the application of WeChat follow-up to provide medical and psychological support to caregivers of infants with leukemia during home-based care could effectively reduce their anxiety and care burden and improve their quality of life (9). However, according to a literature search, there is no report of WeChat being applied for the follow-up management of BPD infants after discharge. This study aimed to evaluate the impact of WeChat follow-up management on the improvement of the psychological distress, care burden, and quality of life of parents of infants with BPD in the context of the pandemic.

## Methods

This was a retrospective cohort study conducted in a hospital in southern China. This study was approved by the ethics committee of the hospital. Since this study was retrospective, the ethics committee approved the waiver of the requirement for informed consent. The inclusion criteria were as follows: (1) infants diagnosed with moderate or above bronchopulmonary dysplasia according to the BPD classification of the National Institute of Child Health and Human Development (NICHD) (10); (2) parents who were the primary caregivers for their children; (3) parents with smartphones and a certain level of education, who were capable of using WeChat for learning and communication, including reading healthcare-related articles on BPD sent by doctors through the WeChat platform; (4) parents who completed the follow-up within 3 months after discharge and who could complete the corresponding scale. The exclusion criteria were as follows: (1) patients with other serious diseases or chronic diseases; (2) patients who required surgical treatment; and (3) parents who had severe social and psychological problems and language communication barriers.

The sample size was calculated as follows: According to the differences in the psychological distress and quality of life scores between the two groups in the preliminary experiment results, we assumed an *α *= 0.05, a *β *= 0.2, and a medium effect size, and we determined that each group needed at least 50 participants. Assuming a 10% loss to follow-up rate, the total sample size required was set as 110 (55 per group).

A total of 112 parents of infants with BPD who met the inclusion criteria were included in this study from January 2016 to January 2022. Related data were extracted from the outpatient medical record system. According to different follow-up methods, these patients were classified into the WeChat group and the routine group. Parents of infants in the routine group received standard discharge health education from the hospital, including leaflets with popularized knowledge and education on BPD and home care precautions. Doctors and nurses conducted one-on-one health education on postdischarge precautions for parents of infants with BPD. In the WeChat group, in addition to the standard discharge health education, the doctors also asked the primary caregivers to join a special WeChat group. The health follow-up mediated by WeChat was mainly divided into three parts: (1) A total of 4–5 popular papers on health education about BPD and popularized BPD-related family oxygen therapy and nutritional support knowledge were pushed through the WeChat platform every week. Parents could view and study the documents at any time that was convenient for them. (2) Family members were assisted in developing personalized nutrition support and family oxygen therapy strategies according to the specific situation of the infants. (3) A fixed group of medical staff were available to the parents from 19:00 to 21:00 every day. If the infants' parents were found to have severe anxiety, the doctor would add a separate private WeChat group to provide one-on-one emotional counseling and support.

The parents were asked to complete the Chinese version of the Social Demographic scale, Depression, Anxiety and Stress Scale-21 (DASS-21), Zarit Caregiver Burden Interview (ZBI), and WHOQOL-BREF at discharge, and the relevant medical data of the infants were also collected. In addition, the DASS-21 scale, ZBI and WHOQOL-BREF were completed by the parents as an outpatient review during the 3-month follow-up. At the same time, the incidence of adverse events during home care was analyzed, including pneumonia, cholestasis, feeding intolerance, jaundice, necrotizing enterocolitis, renal insufficiency, asphyxia, etc. All information in this study was collected for research purposes and was strictly confidential. All the data needed for the study were collected by a particular research assistant.

The Depression Anxiety Stress Scale-21 (DASS-21): We measured depressive, anxiety, and stress symptoms in all primary caregivers using a self-report 21-item scale, a short version of the 42-item standard DASS that has been evaluated for reliability and validity in China (11). The scale had three subscales to assess depression, anxiety, and stress. Participants scores items using a 4-point (0–3) Likert-type scale (ranging from “does not apply to me at all” to “applies to me most or all of the time”). The higher the score is, the higher the severity of depression, anxiety, and stress symptoms. Each subscale receives a score between 0 and 21, with an overall score between 0 and 63. A score of more than 4 for depression, more than 3 for anxiety, and more than 7 for stress indicates mild or higher levels of depression, anxiety, or stress.

The Zarit Caregiver Burden Interview (ZBI) has been confirmed to be reliable and effective in China (12). It consists of 22 items rated on a 5-point scale: Questions 1 to 21 are related to the burden of care experienced by caregivers in various settings, and the last item is related to perceived difficulties in holistic care. Scores range from 0 to 88, with a higher score indicating a “heavier” burden caused by the experience. While there is no universal cutoff point, a study that included Japanese patients with multiple diseases and demographic data reported a cutoff of 24. Using this study, we defined a score of 24 or above as indicating a significant care burden.

The reliability and validity of the WHOQOL-BREF have also been confirmed in China (13). The tool is a 26-item version of the WHOQOL-100 questionnaire that provides a quick evaluation tool for health-related functions in four domains. It is a reliable and essential tool. The WHOQOL-BREF covers four areas: physical health, mental health, social relations, and environment. The remaining two items are related to the overall perception of QOL (Q1) and satisfaction with health (Q2). The WHOQOL-BREF is rated on a 5-point Likert-type scale ranging from 1 (strongly agree) to 5 (strongly disagree), and the average score of responses on each subscale give an overall HRQOL score. A higher score indicates a better quality of life.

## Statistical analysis

Data are expressed as the mean and standard deviation (SD) for quantitative variables and the frequency and percentage (%) for qualitative variables. In this study, skewness and kurtosis coefficients were used to analyze whether the data were normally distributed. If the data conformed to a normal distribution after normality testing, an independent sample *T* test was used. If the data did not conform to a normal distribution, a nonparametric test (Mann‒Whitney test) was used for comparison analysis. Categorical variables between the two groups were analyzed using the *χ*^2^ test or Fisher's test. The data were treated using the software IBM SPSS statistics, version 22. A significance level of *P* < 0.05 was used for the statistical test. In this study, two-tailed tests were used for statistical analyses.

## Results

A total of 112 eligible parents were included in this study, and 11 were lost to follow-up. The final analysis included 51 parents in the WeChat group and 50 parents in the routine group. The general information about all infants and their parents is shown in Table 1, and there was no statistically significant difference between the two groups (*P* > 0.05).

**Table 1:** Comparison of demographic characteristics of infants and their parents between the two groups.

	The WeChat group	The routine group	*P* value
Male gender (%)	30 (58.8)	32 (64)	0.684
Gestational age (w)	27.7 ± 1.5	28.1 ± 1.9	0.182
Birth weight (kg)	1.13 ± 0.12	1.18 ± 0.24	0.201
Age at admission (m)	2.8 ± 0.7	2.9 ± 0.8	0.712
Respiratory support (%)	16 (31.4)	14 (28)	0.829
Tracheostomy (%)	0 (0)	0 (0)	–
Parents’ age (year)	30.9 ± 6.5	32.5 ± 7.1	0.258
Female of caregiver	42 (82.4)	39 (78)	0.625
Total care time, m	2.5 ± 0.5	2.4 ± 0.4	0.180
Parents’ educational level			
Below senior high school	6	4	0.807
High school	14	17	
Junior college	19	16	
Bachelor degree or above	12	13	
Family income			
Low income	9	7	0.853
Middle income	27	25	
High income	14	16	
Missing	1	2	
Living environment			
Rural areas	20	17	0.681
City	31	33	

There was no significant difference in the scores of the DASS-21, ZBI, and WHOQOL-BREF between the two groups at discharge. After comparing the DASS-21 and ZBI of the two groups at the 3-month follow-up and at discharge, it was found that the anxiety score and stress score of the WeChat group were significantly lower than those of the routine group. The care burden score of the WeChat group was also significantly lower than that of the routine group (*P* < 0.05) (Table 2).

**Table 2:** Comparison of the scores of DASS-21 and ZCBI at discharge and three months later were compared between the two groups.

	The WeChat group	The routine group	*P* value
At the time of discharge			
Depression score	3.2 ± 1.1	3.4 ± 0.9	0.321
Anxiety score	4.1 ± 1.5	3.9 ± 1.6	0.523
Stress score	5.5 ± 1.3	6.1 ± 2.0	0.116
Follow-up after 3 months			
Depression score	8.4 ± 3.7	9.0 ± 4.2	0.459
Anxiety score	5.7 ± 1.9	7.2 ± 2.6	0.002
Stress score	8.5 ± 2.7	10.8 ± 4.1	0.001
Zarit Caregiver Burden Interview (ZCBI)	50.5 ± 11.7	57.4 ± 13.8	0.007

We analyzed the WHOQOL-BREF scores of the parents at the 3-month follow-up. The scores in the psychological and social relations fields in the WeChat group were significantly higher than those in the routine group (*P* < 0.05). There was no statistically significant difference in the physiological field and environmental field scores between the two groups. The total score of the WHOQOL-BREF in the WeChat group was significantly higher than that in the routine group (*P* < 0.05) (Table 3). A comparative analysis of adverse events between the two groups during the 3-month follow-up showed that the incidence of pneumonia in the WeChat group was significantly lower than that in the routine group (*P* < 0.05). There were no significant differences in the incidence of cholestasis, feeding intolerance, jaundice, necrotizing enterocolitis, renal insufficiency, and asphyxia between the two groups (*P* > 0.05) (Table 4).

**Table 3:** Comparison of the scores of WHOQOL-BREF scale between the parents of the two groups at 3 months follow-up.

	The WeChat group	The routine group	*P* value
Physiological field	12.5 ± 2.1	11.7 ± 2.6	0.132
Psychological field	15.6 ± 3.8	13.7 ± 3.2	0.007
Social relations field	16.3 ± 4.7	14.1 ± 3.0	0.005
Environmental field	11.6 ± 3.9	12.9 ± 3.5	0.085
Total score of quality of life	56.1 ± 7.0	52.4 ± 6.9	0.010

**Table 4:** Comparison of complications 3 months follow-up between the two groups.

	The WeChat group	The routine group	*P* value
Pneumonia	9	18	0.045
Cholestasis	1	2	0.617
Feeding intolerance	5	8	0.389
Jaundice	9	11	0.621
Necrotizing enterocolitis	0	1	–
Renal insufficiency	0	0	–
Asphyxia	0	0	–
Death	0	0	–

## Discussion

This was a retrospective cohort study on the influence of follow-up medical support provided through WeChat on the psychological distress, care burden, and quality of life of parents of infants with BPD receiving home care. Our study found that the psychological burden of parents of infants with BPD generally increased in the process of home care. The depression, anxiety, and psychological pressure showed varying degrees of elevation in these parents. In addition, we found that WeChat follow-up support could significantly relieve the anxiety and psychological stress, reduce the care burden, and improve the quality of life of parents of infants with BPD.

In this study, we found that BPD patients’ state changed from being dominated by professional nurses in the NICU to being dominated by parents in the family home. Due to the lack of experience, the parents did not understand the related knowledge, which caused significant psychological distress for them and led to depression and anxiety, so these parents were under heavy psychological pressure. This finding was similar to the result from Christine et al., whose study found that mothers of infants with BPD had poorer sleep quality and a higher psychological burden than the general population (14). This result might be caused by the fact that most premature infants with BPD are young, stunted, and immature regarding various organ systems and are prone to various emergencies during in-home oxygen therapy and feeding (15). Most of the parents did not have this experience; therefore, the transition process was very tough. Sharon et al. also confirmed this result, and their research showed that the longer the duration of providing nursing care for infants with BPD, the worse the physical conditions, worries, and daily lives of the parents, presenting a trend of gradual decline (16).

We found that WeChat provided routine popular disease knowledge, solved problems in a timely manner, and provided timely relief of psychological distress. It could significantly relieve the anxiety and psychological pressure, reduce the care burden, and improve the quality of life of parents of infants with BPD. The parents needed to learn much about BPD-related disease, but they did not have an appropriate way to learn about this disease. Studies have shown that a lack of knowledge about the disease significantly aggravates parents' anxiety (17, 18). The care measure time needs to be adjusted according to the situation of the infant with BPD in the home care process, and the frequency was much higher than that of other diseases. However, medical resources were relatively limited and unevenly distributed in our district, and some of the parents who lived in areas at the grassroots level could not adjust to the patient's care strategy in a timely manner and were also unable to solve their problems promptly, which led to heavy psychological burden or even collapse for a long time. The heavy psychological burden further reduces quality of life. WeChat, which has a high popularity rate in China, could provide a good platform for knowledge popularization. It could help parents more calmly face various situations and relieve their poor psychological conditions. In addition, the incidence of pneumonia in the WeChat group was significantly lower than that in the routine group. This finding might be attributed to the fact that WeChat follow-up education helped parents better grasp nursing techniques for infants with BPD and enabled them to receive timely professional assistance when encountering relevant special circumstances, thereby reducing the occurrence of pneumonia to some extent. Reducing complications could also ease the psychological burden on parents.

With the development of modern medical technology and concepts, current treatment strategies not only focus on patients’ conditions and quality of life but also pay increasing attention to caregivers' quality of life. Caregivers providing long-term care for patients with chronic diseases have seriously impacted quality of life. Many previous studies have shown that parents of infants with chronic diseases have significantly reduced quality of life (19, 20). Lee and his colleagues' findings suggested that positive social support could significantly improve caregivers' quality of life (21). This point was also suggested by our study. We found that the quality of life score of the WeChat group was significantly higher than that of the routine group, which also showed the effectiveness of medical support provided by the WeChat platform. This study provides effective evidence to improve the psychological distress and quality of life of parents of infants with BPD and confirms that WeChat follow-up could be well applied to parents of infants with BPD, which is worth promoting.

Although many influencing factors were considered in the design process of this study, there were still some limitations. First, in this study, parents were required to have a certain level of education to better learn related disease knowledge pushed through WeChat, which might lead to the WeChat management mode not being suitable for every parent of infants with BPD. Second, there were no infants with tracheostomy or tracheostomy + ventilation dependance in this study, which might have affected the accuracy of the findings. Third, this study failed to evaluate the effect of long-term transitional care provided through WeChat on infants with BPD because only short follow-up records were used. Finally, this was a single-center retrospective study and could not prospectively collect targeted data.

## Conclusion

WeChat follow-up management is helpful to decrease the anxiety and stress, reduce the care burden, and improve the quality of life of parents of infants with BPD receiving in-home care.

## Data Availability

The raw data supporting the conclusions of this article will be made available by the authors, without undue reservation.
